# Phasic stimulation in the nucleus accumbens enhances learning after traumatic brain injury

**DOI:** 10.1093/texcom/tgac016

**Published:** 2022-04-09

**Authors:** Joshua P Aronson, Husam A Katnani, Anna Huguenard, Graham Mulvaney, Edward R Bader, Jimmy C Yang, Emad N Eskandar

**Affiliations:** 1 Department of Neurosurgery, Harvard Medical School, Massachusetts General Hospital, 55 Fruit Street, Boston, MA, 02114, United States; 2 Section of Neurosurgery, Department of Surgery, Dartmouth-Hitchcock Medical Center, One Medical Center Drive, Lebanon NH, 03756, United States; 3 Department of Neurological Surgery, Albert Einstein College of Medicine, 1410 Pelham Parkway South, Rose F. Kennedy Center, Bronx, NY, 10461, United States

**Keywords:** deep brain stimulation, learning, microstimulation, nucleus accumbens, traumatic brain injury

## Abstract

Traumatic brain injury (TBI) is a significant cause of morbidity and mortality worldwide. Despite improvements in survival, treatments that improve functional outcome remain lacking. There is, therefore, a pressing need to develop novel treatments to improve functional recovery. Here, we investigated task-matched deep-brain stimulation of the nucleus accumbens (NAc) to augment reinforcement learning in a rodent model of TBI. We demonstrate that task-matched deep brain stimulation (DBS) of the NAc can enhance learning following TBI. We further demonstrate that animals receiving DBS exhibited greater behavioral improvement and enhanced neural proliferation. Treated animals recovered to an uninjured behavioral baseline and showed retention of improved performance even after stimulation was stopped. These results provide encouraging early evidence for the potential of NAc DBS to improve functional outcomes following TBI and that its effects may be broad, with alterations in neurogenesis and synaptogenesis.

## Introduction

Traumatic brain injury (TBI) is a major cause of morbidity and mortality, with an estimated worldwide incidence of 69 million cases annually (Dewan et al. 2018). While current medical and surgical management has improved survival, treatments that improve functional outcome are lacking and a large proportion of patients are left with significant disabilities (He et al. 2004; Scafidi et al. 2010). Decompressive craniectomy can reduce mortality but has not been shown to significantly improve functional outcomes (Cooper et al. 2011; Hutchinson et al. 2016); pharmaceutical trials, usually performed in the acute period following TBI, are yet to demonstrate a clear functional benefit (Skolnick et al. 2014); and physical/behavioral rehabilitation is limited in its ability to accelerate recovery (Vink and Nimmo 2009). Accordingly, there is a pressing need for novel treatments targeted at improving functional outcomes following TBI.

Advances in deep brain stimulation (DBS) have created enthusiasm for its use as a therapy for TBI. DBS is effective at treating movement disorders, such as Parkinson’s disease, and refractory epilepsy via stimulation of the anterior nucleus of the thalamus (Fisher et al. 2010; Salanova et al. 2015), demonstrating that DBS can modulate brain networks. Recent experimental evidence has also shown that precisely timed DBS of the striatum can enhance learning (Katnani et al. 2016; Bick et al. 2019). However, only a handful of clinical studies have investigated the efficacy of DBS for TBI to date (Tsubokawa et al. 1990; Schiff et al. 2007; Rezai et al. 2016). These studies have typically involved patients who are severely impaired (for example, minimally conscious) and have yielded mixed results. Accordingly, there remains a need to understand the potential of DBS for improving functional outcomes in individuals with moderate impairments and using temporally precise stimulation paradigms to target other cognitive deficits.

The nucleus accumbens (NAc) is a dopaminergic structure that has rich cortical and subcortical connectivity (Joel and Weiner 2000). This broad connectivity allows the NAc to modulate regions in the frontal lobes, midbrain, and brainstem to drive cognitive function and mediate behavior (Zahm 2000). Accordingly, the NAc has been proposed as a substrate for reinforcement learning (RL) (Kelley et al. 1997). In keeping with this framework, lesions of the NAc result in learning deficits (Annett et al. 1989) and neurophysiological studies demonstrate that signal processing in the NAc conforms with RL models (Joel et al. 2002). Specifically, phasic firing in the NAc exhibit signals consistent with a reward prediction error, a key component of RL models (Day et al. 2007; Wassum et al. 2013). These phasic patterns of activity have been linked to activation in the prefrontal cortex, suggesting a phenomenon across the corticostriatal system that, when modulated, can promote neural plasticity and behavioral change through dopaminergic signaling (Reynolds et al. 2001; Rinaldi et al. 2012). The NAc is a promising target for translational research, particularly in the setting of DBS in which stimulation is utilized in an attempt to modulate the dopaminergic interface between the limbic system and motor system for treatment of neurological disorders (Di Chiara et al. 2004; Holtzheimer and Mayberg 2011; Albaugh et al. 2016). Furthermore, studies have revealed that dopamine release by the NAc is altered following TBI (Chen et al. 2017), suggesting that targeting the NAc may be particularly beneficial. With converging evidence suggesting that the NAc can drive corticostriatal activity to induce behavioral change and plasticity, we hypothesized that task-matched stimulation of the NAc could enhance rehabilitation following TBI.

Here, we investigated the potential of a targeted, task-matched DBS therapy targeted to the NAc in which precisely timed stimulation augmented RL during a visuomotor spatial learning task in a rodent model of TBI. We demonstrate, for the first time, that NAc DBS can enhance learning following TBI. We further demonstrate that animals receiving DBS exhibited greater behavioral improvement and enhanced neural proliferation compared to controls. Treated animals recovered to an uninjured behavioral baseline and showed long-term memory benefits of learned behaviors even after cessation of stimulation. These results suggest that NAc DBS has a strong potential to improve functional outcomes following TBI and that its effects may be broad, with alterations in neurogenesis and synaptogenesis.

## Materials and methods

All surgical procedures complied with the Guide for the Care and Use of Laboratory Animals published by the National Institutes of Health (DHEW publication NIH 85-23-2985), and the protocols were approved by the Massachusetts General Hospital Institutional Animal Care and Use Committee.

### TBI model

Adult (10-week-old) male C57BL/6 mice were anesthetized with isofluorane and were mounted on a stereotactic frame (Kopf Instruments, Tujunga, CA). A 10-mm midline scalp incision was made, and a 3.5-mm right parietal craniotomy was made bordering the coronal suture anteriorly and the sagittal suture medially. An electromagnetic impactor (Leica Biosystems, Buffalo Grove, IL) with 3-mm diameter tip was positioned flush with the dura. Injury was induced using impactor velocity of 5.2 m/s, depth of 2.65 mm, and dwell time of 100 ms. The bone flap was replaced, and the incision closed with interrupted absorbable sutures. Controlled cortical impact resulted in complete unilateral destruction of the hippocampus (HPC; Supplementary Fig. 1a). TBI severity was classified as moderate and a wire grip test (Supplementary methods; Supplementary Fig. 1b) was utilized to assess motor function postinjury (Bermpohl et al. 2006) along with cage behavior to assess signs of abnormal behavior. Mice were divided into 3 study groups: craniotomy without cortical impact (uninjured control), mice with cortical impact without stimulation (untreated control), and mice with cortical impact and stimulation (intervention).

### DBS electrode implant

Seven days after cortical impact, all mice were implanted with a 3-contact, concentric, miniature DBS electrode (Fred Haer Corporation, Bowdoin, ME). Electrodes had a 0.1-mm distal contact, a second 0.1-mm contact located 1.35 mm proximal along the shaft, and a ground contact located just below a 3-pin connector. Mice were anesthetized and positioned in the stereotactic frame. Implant coordinates were chosen to position the distal contact in the NAc and the proximal contact in the caudate, with the ground contact resting just below dura (from bregma, 1.10 mm anterior, 1.35 mm lateral, 3.82 mm ventral; Supplementary Fig. 1c). A 0.2-mm right frontal craniectomy was made at the planned entry site. The electrode was cemented in place using acrylic dental cement. Animals were given 1 week to recover.

### Stimulation parameters

In the stimulation group, the NAc (and/or caudate) contacts were used as the cathode and a subdural contact was used as the anode. Stimulation was delivered as constant current with symmetric, biphasic, cathodic leading square wave pulses. High-frequency stimulation parameters were set to 50 μA, 130 Hz, and 80 μs pulse width per phase. For low-frequency stimulation, frequency was changed to 50 Hz. Bursting stimulation in the real-time place preference assay utilized 500-ms trains of the high-frequency stimulation parameters with 500 ms between trains. For the stimulation group, 5 s of stimulation was delivered 5 s after arriving on the platform. In the early NAc stimulation group, 5 s of stimulation was delivered while the animal was facing the directional cue, prior to release into the water. For retention testing, animals were rested for 7 days and were retested without any further stimulation for an additional 5 consecutive days.

### Morris water maze

Visuospatial associative learning was assessed using a Morris water maze (MWM) paradigm (Morris 1984). A white pool (120 cm diameter, 100 cm deep) was filled with water to 70 cm depth. In the northwest quadrant, a round, clear plexiglass platform that was 10 cm in diameter was positioned 1 cm below the water surface. Testing was conducted 2 weeks after injury in the subacute phase, when the brain is in a state of recovery and injury effects have stabilized, allowing for an evaluation of stimulation as a rehabilitation treatment. Injured animals were split into 2 groups, treated and untreated, with treated animals receiving stimulation with parameters analogous to clinical biphasic high-frequency DBS (−50 μA, 130 Hz, 80 μs per phase). During the task, treated mice received 5 s of stimulation upon reaching and resting on the platform, a strategy designed to reinforce goal location while animals observe their environment, registering their position on the platform with reference to the abstract cues and the larger room. Learning performance was assessed using mean escape latency (time taken for the mouse to arrive at the platform).

**Fig. 1:**
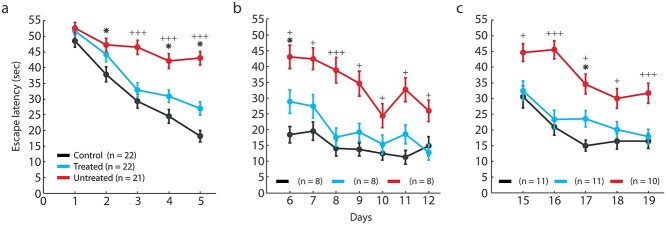
Stimulation improved spatial memory performance. a) Escape latency decreased in all groups across the first 5 days of testing. Treated animals (cyan) displayed significantly better performance than untreated animals (red) after 2 days of testing. A significant group effect was identified (*P* < 0.001). A significant difference between control and injured animals was found on and after 2 days (*P* < 0.05 for all; except day 3 between control and treated animals). A significant difference between treated and untreated animals was identified on day 3 (*P* < 0.05). b) Testing for a portion of animals in each group (*n* = 8 animals) was continued for 12 days. Escape latency performance for treated animals began to overlap with control animals (black) after 6 days, eventually reaching steady state at nearly the same baseline. A significant group effect was identified (*P* = 0.001). A significant difference between uninjured control and treated animals was found only on day 6 (*P* < 0.05). Untreated animals had a greater escape latency when compared to both uninjured control and treated animals across all days (*P* < 0.05). c) After the first 5 days of testing, a portion of animals in each group (uninjured control *n* = 11 animals; treated n = 11 animals; untreated, *n* = 10 animals) were given 10 days of rest before being retested. Treated and uninjured control animals performed similarly, with minimal regression in performance. A significant group effect was identified (*P* < 0.001). A significant difference between uninjured control and treated animals was found only on day 17 (*P* < 0.05). Untreated animals demonstrated significantly longer latency compared with both control and treated animals across all days (*P* < 0.05). ^*^ indicates statistical significance between both TBI cohorts and the control animals. ^+^indicates statistical significance of untreated animals from both treated and control animals. Values are mean ± s.e.m., ^*^ and ^+^*P* < 0.05, ^+++^*P* < 0.001. *P*-values for group effects calculated using repeated measure ANOVA; post hoc pairwise Tukey’s tests were used to assess for differences between groups using a 2-tailed *t*-test incremented comparison on individual days.

Mice performed 4 trials per day, once at each of the 4 starting locations marked with abstract cues (north, south, east, and west) and were placed in the pool facing the cue mounted on the wall of the pool. Mice were tethered by their headcaps to an overhead wire and were given a maximum of 60 s to find the platform. If the mouse failed to reach the platform by 60 s, it was placed on the platform by the experimenter and allowed to remain there for 20 s. For 12- and 19-day testing (Fig. 1b and c, respectively), 2 different groups of mice were tested, with results for days 1–5 pooled. At the conclusion of the 5- or 12-day testing period, a probe trial was performed in which mice were placed in the tank with the platform removed and the latency in the target quadrant was measured. Behavioral data (search path, latency, distance, etc.) were captured using digital video and a custom automated tracking system designed in MATLAB (Mathworks, Natick, MA). Code is available on reasonable request from the corresponding author.

### Real-time place preference

To evaluate for a hedonic or aversive response to stimulation in the NAc, a real-time place preference assay was utilized. Mice were placed in a 20 × 20-cm square chamber bisected by a wall with a 3-cm door and were allowed to range freely for 30 min. Constant or bursting stimulation was delivered when mice were located on the stimulation-paired side of the chamber. Automated video tracking was used to record time located on each side.

### Immunohistochemistry

On the first 2 consecutive days of MWM testing, animals were injected with 100 mg/kg BrdU. Six hours after the fifth day of MWM testing, groups of animals were anesthetized with isofluorane and underwent transcardiac perfusion with 10 mL of phosphate-buffered saline followed by 10 mL of 4% paraformaldehyde. The brains were extracted, and green tissue dye was applied to the probe extract site. Brains were postfixed in 10% formalin for 48 h, bisected sagittally, and placed into formalin before processing.

Both hemispheres of the fixed and the processed brains were paraffin-embedded and sectioned into ~25-μm thick sagittal slices with 2 sections collected every 50 μm. Deparaffinized slides underwent citrate buffer antigen retrieval and were incubated for 24 h at 4°C with the following primary antibodies: rat monoclonal anti-BrdU (1:50; Abcam AB6326), goat polyclonal anti-Doublecortin (1:100; Santa Cruz SC-8066), rabbit polyclonal anti-NeuN (1:500; Abcam AB104225), rabbit polyclonal anti-synapsin-1 (1:100; Abcam AB64581), and goat polyclonal anti-GAP 43 (1:50; Santa Cruz SC-7457). Slides were treated with the following Alexa Fluor conjugated secondary antibodies for 1 h at room temperature: donkey anti-rat IgG H&L 488 (1:200; Life Technologies A-21208), donkey anti-rabbit IgG H&L 647 (1:200; Life Technologies A-31573), donkey anti-goat IgG H&L 555 (1:200; Life Technologies A-21432), and donkey anti-rabbit IgG H&L 488 (1:200; Life Technologies A-21206). Slides were mounted with a DAPI counterstain medium (Vectashield).

### Imaging and quantification

Cell-counting and identification of colabeled cells were performed after capturing images on a confocal microscope (Sp8; Leica). Analysis was blind to the behavioral group and behavioral results. Cell counting was performed on 25-μm thick slices under a 40× objective in 4 regions of interest bilaterally: hippocampus, subventricular zone (SVZ), striatum, and anterior rostral migrating stream (RMS). Each animal yielded 6 stained sections of each antibody combination. The total number of NeuN^+^/BrdU^+^ cells and DCX^+^/BrdU^+^ cells were hand-counted for each collected section based on fluorescence while analyzing along the 3D stack. The number counted was totaled across the 6 sections and then averaged with animals of the same subgroup. For synapsin and GAP-43 intensity labeling, upright fluorescence images were captured in the same regions of interest using an Arcturas Veritas microscope. Histograms of pixel intensity for each image were generated using ImageJ software and the average pixel intensity was calculated using binned intensities by pixel and averaged across the 6 stained sections. Negative control slides, produced following identical staining protocol as above without application of the primary antibody, were used to subtract background staining in each region of interest for each subgroup.

### Real-time polymerase chain reaction

For gene expression analysis, the SVZ was excised and flash frozen. Total RNA was isolated from cell pellets using Trizol Reagent (Invitrogen) and the concentration was determined using both a BioPhotometer spectrophotometer (Eppendorf) and RNA 6000 kit (Agilent). cDNA was then generated from 1 μg of total RNA using Superscript II Reverse Transcriptase (Invitrogen). Quantitative real-time PCR reactions were run in 25-μL volumes on a CFX96 Fast Real-Time PCR Detection System (BIO-RAD) using iQ SYBR Green Supermix (BIO-RAD). Primers were designed using Primer-Blast (NCBI) and PrimerQuest Tool (Integrated DNA Technologies) and were validated for effective amplification without the interference of primer dimer up to a minimum 38 cycles. The following genes were evaluated: Nestin, Sox-2, Dcx, BDNF, Bmi-1, GAPDF (Supplemental methods). All qPCRs were performed in biological and technical triplicates. Data were analyzed using MATLAB and Microsoft Excel. GAPDH was used as an endogenous normalization control and the fold expression relative to GAPDH was determined by the ΔΔCt method. Relative gene expression between treated and untreated injured animals was evaluated using the ${2}^{-\Delta \Delta \mathrm{Ct}}$ method (Livak and Schmittgen 2001). All values were subtracted by 1 to show fold expression between the 2 cohorts from 0.

### Statistical analysis

All distributions passed tests for normality (Kolmogorov–Smirnov) and for equal variance (Levene Median) unless noted differently. Repeated-measures ANOVA was used to test for escape latency group effects, with days as the repeated measure and escape latency as the dependent variable. Post hoc pairwise Tukey’s tests were then used to assess for differences between control and treated animals using a 2-tailed *t*-test incremented comparison on individual days. Path efficiency was calculated utilizing the following equation:

$$ 1-\frac{D-O}{D+O}, $$

where *D* is the traveled path length and *O* is the optimal path length. Distributions for each group, as well as the distribution of slope coefficients for each group output from the linear regression of path efficiency across days for each animal, were compared using a 2-tailed, paired Student’s *t*-test. Learning was modeled with a log-linear learning model:

$$\begin{eqnarray*} \ln \left(\mathrm{l}{\mathrm{atency}}_{\mathrm{mtd}}\right)\!\!\!&={\alpha}_d+\beta 1\left({\mathrm{day}}_t\right)+\beta 2\left({\mathrm{stim}}_m\right)\nonumber\\ &\quad+\,\beta 3\left({\mathrm{day}}_t\ast{\mathrm{stim}}_m\right)+{\epsilon}_{\mathrm{mtd}}. \end{eqnarray*}$$

A 2-tailed, paired Student’s *t*-test between respective groups was used to assess for a hedonistic response to stimulation by comparing time spent in each side. To compare immunofluorescence labeling intensity, a Mann–Whitney rank sum test was used to compare average pixel intensity between groups (all were nonnormally distributed). When comparing fold expression of primers using RT-qPCR, fold expression for each animal in their respective cohort was first normalized relative to their own GAPDH levels via the ΔΔCt method. Paired Student’s *t*-tests were then used for each normalized primer distribution.

## Results

### DBS following brain injury enhances behavioral performance

All animals were first tested across 5 days during which the time taken for mice to reach the platform decreased in all groups (Fig. 1a). Animals which received stimulation showed greater improvement in behavioral performance than untreated controls, with significantly shorter escape latencies after 2 days of testing (Fig. 1a, *P* < 0.001). Following 5 days of testing, each group was split, with a cohort of mice continuing behavioral testing and the remaining mice receiving a 10-day rest period. Continued testing was performed to establish the maximal improvement in spatial learning for each group. After day 6, escape latency performance of treated animals was not significantly different from control animals, and both groups reached a similar performance plateau (Fig. 1b). Furthermore, the distribution of learning rate coefficients (control: −0.1097, treated: −0.1099, untreated: −0.06) revealed that treated mice learned at a significantly faster rate and to a greater level than untreated mice and approximately to the same extent as control mice.

Mice that received a 10-day rest period after initial testing were retested on the behavioral task with and without the same platform location (animals which previously received stimulation did not receive any further stimulation). On day 1 of the task, untreated injured animals exhibited regression of task performance with escape latency reverting to a near-naive state, while previously stimulated and control animals showed only moderate loss in performance with quick recovery to their posttraining performance plateau (Fig. 1c). There was no significant difference in performance between mice that received stimulation and uninjured control mice (Fig. 1c).

**Fig. 2:**
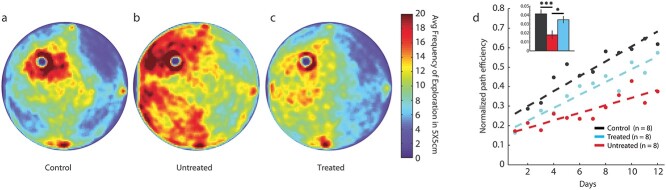
Stimulation increased search pattern efficiency. a–c) Top-down view of spatial exploration taken across 12 days of testing for the control, untreated, and treated animals (*n* = 8 animals per group), respectively. Control animals displayed focal search patterns near the hidden platform, while untreated animals showed distributed exploration of the entire space. Treated animals displayed less-dispersed search patterns than untreated animals with exploration targeted near the hidden platform. Scale bar indicates frequency of visiting a 5X5 cm area. d) Path efficiency increased in all groups across 12 days of testing with the rate of rise for control (black) and treated (cyan) animals being greater than untreated animals (red). Values are mean ± s.e.m., ^^*^^P < 0.05, ^^*^^*^^*^^P < 0.001. *P*-values calculated using a 2-tailed, paired Student’s *t*-test.

Efficiency in path exploration was evaluated across the 12 days of continuous testing to visualize the escape latency improvement. Control and untreated injured mice exhibited different search patterns: Control animals exhibited a focused search near the platform while untreated animals showed distributed search patterns that encompassed most regions of the maze (Fig. 2a and b). Treated mice demonstrated more distributed search patterns than control animals but targeted the quadrant near the platform (Fig. 2c). Linear regression of path efficiency for each group (Fig. 2d), calculated from the search patterns of each day, revealed that control animals and treated animals improved across days of testing and at a similar rate (control: 0.038, treated: 0.033). Untreated animals did not show the same rate of improvement in path efficiency scores and derived rates of improvement that were significantly worse than treated and control animals (untreated: 0.019). There were no differences in average velocity between groups.

### DBS in the NAc did not induce hedonic a response

**Fig. 3:**
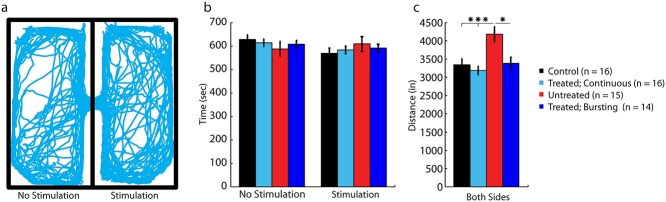
Task stimulation parameters did not induce a hedonic response. a) Real-time place-preference location plot from a representative treated animal, with continuous stimulation on the stimulated-paired side, showing position over the course of a 20 min session. b) Uninjured control animals (black, *n* = 16 animals), treated animals, receiving continuous (cyan, *n* = 16 animals) or bursting (blue, *n* = 14 animals) stimulation, and untreated animals (red, *n* = 15 animals) spent approximately the same amount of time on the nonstimulated and stimulated sides, demonstrating that treated animals did not have a preference to stimulation. c) Untreated animals traveled a significantly greater overall distance during the 20-min session, indicating a potential hyper-active state due to injury. Values are mean ± s.e.m., ^^*^^P < 0.05, ^^*^^*^^*^^P < 0.001. *P*-values calculated using a 2-tailed, paired Student’s *t*-test between respective groups.

The real-time place preference task revealed no significant differences between groups in time spent exploring either side of the environment (Fig. 3b), regardless of the DBS setting, indicating that stimulation did not cause a hedonic response. However, untreated injured mice were found to be hyperactive during the task, traveling a significantly greater distance during exploration (Fig. 3c). This was not observed in treated mice, with no significant difference in the distance traveled compared to uninjured control mice, suggesting a normalization in behavior induced by DBS.

Several other control experiments were conducted to verify the therapeutic benefit of the applied DBS strategy targeted in the NAc. Behavioral testing was repeated with new injury groups, including paradigms in which low frequency stimulation (50 Hz) was used, stimulation was applied continuously during the task, stimulation was applied at a different temporal epoch of the task (during placement in front of a visual cue), and stimulation was applied in the caudate nucleus (Supplementary Fig. 2). Escape latency was also compared between uninjured control animals and uninjured control animals which received stimulation (Supplementary Fig. 3). In each case, no significant differences in behavioral performance were observed.

### Restoring synaptic density

**Fig. 4:**
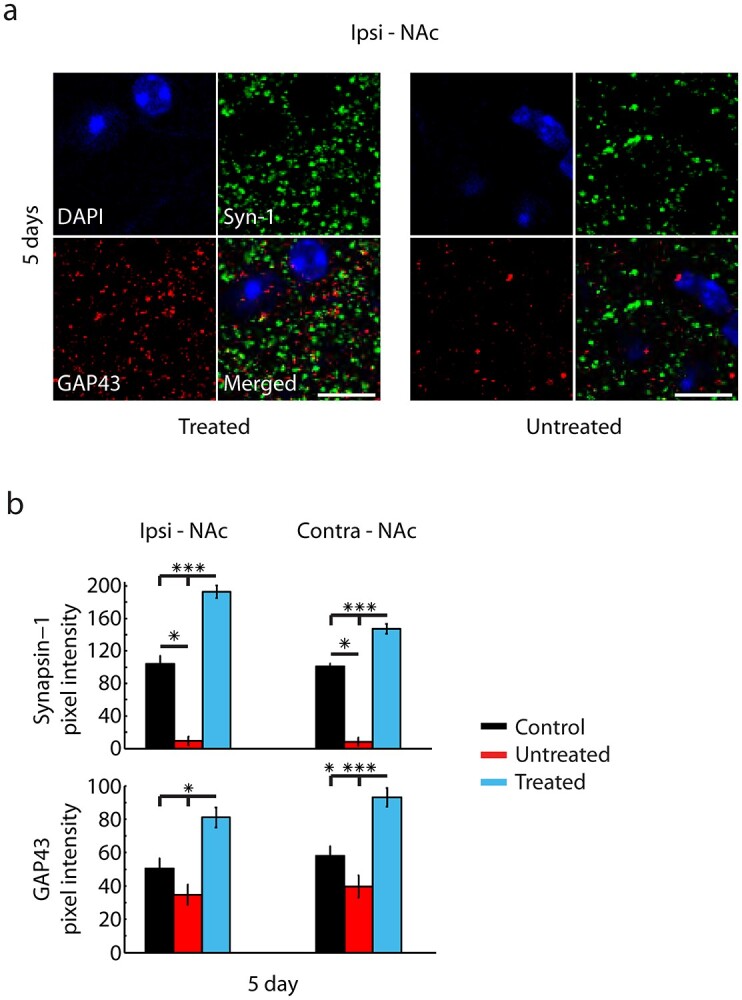
Striatal stimulation restored synaptic density and promoted neuronal outgrowth. a) Representative 40× images of synapsin-1 (green) and GAP43 (red) coexpressed with DAPI (blue) in the ipsilesional NAc for a treated and untreated animal at 5. b) Top: Mean pixel intensity of synapsin-1 labeling in the ipsilesional and contralesional NAc at 5 days for control (black; *n* = 3 animals, 6 sections per animal), untreated (red; *n* = 3 animals, 6 sections per animal), and treated (cyan; *n* = 3 animals, 6 sections per animal) animals. Treated animals showed significantly greater intensity than both control and untreated animals. Control animals showed significantly greater intensity than untreated animals. Scale bars = 10 μm. b) Bottom: Mean pixel intensity of GAP43 labeling in the ipsilesional and contralesional NAc at 5 days for control (black), untreated (red), and treated (cyan) animals. Treated animals showed significantly greater intensity when compared to both control and untreated animals. Values are mean ± s.e.m., ^^*^^P < 0.05, ^^*^^*^^*^^P < 0.001. *P*-values calculated using a Mann–Whitney rank sum test.

**Fig. 5:**
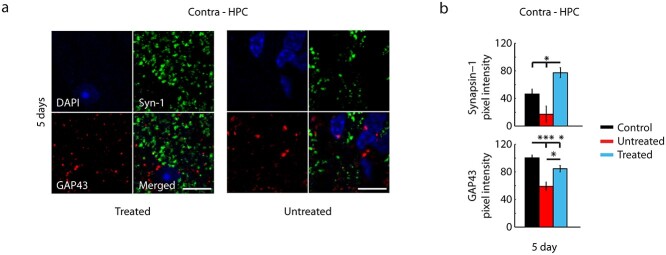
Enhanced synaptic density and neural outgrowth in the hippocampus. a) Representative 40× images of synapsin-1 (green) and GAP43 (red) coexpressed with DAPI (blue) in the contralesional hippocampus for a treated and untreated animal at 5 days. Scale bars = 10 μm. b) Top: Mean pixel intensity of synapsin-1 labeling in the contralesional HPC at 5 days for control (black), untreated (red), and treated (cyan) animals (*n* = 3 animals per group, 6 sections per animal). Treated animals showed significantly greater intensity when compared to control and untreated animals. b) Bottom: Mean pixel intensity of GAP43 labeling in contralesional HPC at 5 days for control (black), untreated (red), and treated (cyan) animals (*n* = 3 animals per group, 6 sections per animal). Control animals show significantly greater intensity than both treated and untreated animals. Treated animals also showed significantly greater intensity than untreated animals. Values are mean ± s.e.m., ^^*^^P < 0.05, ^^*^^*^^*^^P < 0.001. *P*-values calculated using a Mann–Whitney rank sum test.

To evaluate if NAc stimulation altered synaptic function, a form of plasticity that can occur within minutes to hours, we assessed the mean pixel intensity of synapsin-1, a marker of synaptic vesicles implicated in synaptogenesis (Chin et al. 1995; Waites et al. 2005). Analysis was targeted to the NAc and HPC, where synaptic plasticity is pivotal for spatial memory learning (Neves et al. 2008; Rinaldi et al. 2012). Animals from each cohort were sacrificed after the first 5 days of MWM testing. A common trend was found for untreated injured animals, with diminished labeling in untreated mice compared to both treated and uninjured control mice in the NAc (Fig. 4) and HPC (Fig. 5). The ipsilesional NAc of treated mice sacrificed after 5 days of MWM testing showed significantly greater synapsin-1 labeling than control mice, which may be expected since the region was the target site for electrical stimulation (Bear and Malenka 1994; Nguyen et al. 2000). However, this result was also seen in the contralesional NAc and HPC, suggesting a widespread effect of stimulation on spatial memory circuitry.

The mean pixel intensity of GAP43, a marker for neural growth cones associated with plasticity and found during long-term potentiation (Strittmatter et al. 1992; Namgung et al. 1997), was assessed. Untreated mice were found to have diminished levels of GAP43 compared to treated mice in both the NAc (Fig. 4) and HPC (Fig. 5), with no significant difference in labeling when compared to uninjured control mice. However, at day 5, uninjured control mice showed significantly more GAP43 labeling than both treated and untreated mice in the contralesional HPC (Fig. 5b). Treated mice showed significantly more GAP43 labeling when compared to untreated mice in both the NAc and HPC and more labeling in the NAc when compared to uninjured control mice.

### Promoting neuronal precursors

**Fig. 6:**
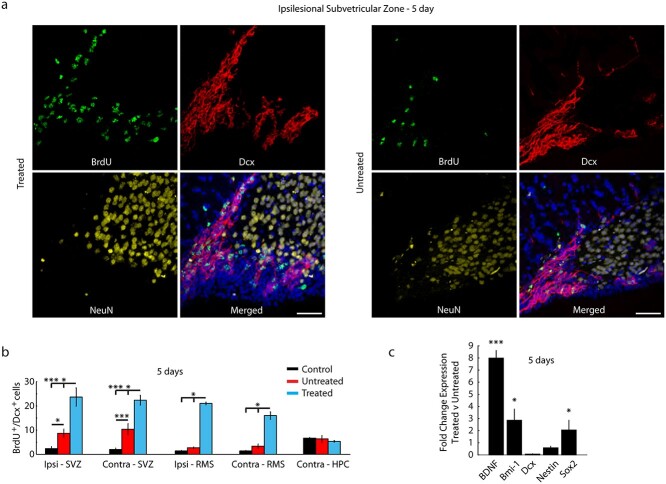
Stimulation increased new neuronal precursors. a) Representative images, taken by a confocal 40× objective, showing ipsilesional SVZ in a treated and untreated animal, respectively, after 5 days of MWM testing. Expression of colabeled dcx (red) and BrdU (green) qualitatively demonstrate the dramatic increase of neural progenitor cells along the SVZ of treated animals. Merged images include DAPI (blue); scale bars = 50 μm. b) Quantitative analysis of the number of colabeled BrdU^+^/dcx^+^ cells found in the ipsilesional SVZ and RMS of untreated (red), treated (cyan), and control (black) animals (*n* = 3 animals, 6 sections per animal). A significantly greater number of cells were found in the ipsilesional SVZ and RMS of untreated animals when compared to control animals, and a significantly greater number of cells were found bilaterally in the SVZ and RMS of treated animals when compared to untreated animals. Additionally, there was an increase in the contralesional SVZ and RMS of treated animals when compared to untreated and control animals. c) Relative comparison of fold expression of 5 primers tested using RT-qPCR in the SVZ of treated (*n* = 8) and untreated (*n* = 6) animals. Treated animals showed significant upregulation of BDNF, Bmi-1, and Sox2 compared to untreated animals. Values are mean ± s.e.m., ^^*^^P < 0.05, ^^*^^*^^*^^P < 0.001. *P*-values comparing pixel intensity calculated using a Mann–Whitney rank sum test; *P*-values comparing fold change expression calculated using a paired Student’s *t*-test.

In the setting of brain injury, innate self-repair mechanisms are activated (Taupin 2006). Accordingly, we investigated whether NAc stimulation generated activity-dependent neurogenesis. Analysis was centered on BrdU+ cells colabeled with double-cortin (DCx), a marker for immature neural stem cells. Evaluation was focused on bilateral labeling in the key regions of interest (HPC, SVZ, and RMS). Animals sacrificed after the first 5 days of MWM testing showed an increase of BrdU incorporation, predominantly in the SVZ (Fig. 6) and RMS (Fig. 6; Supplementary Fig. 4). Both treated and untreated injured mice showed increased labeling in the ipsilesional SVZ compared to control mice.

### Supporting gene expression of neurogenesis

Expression levels of specific genes that have been found to be pivotal in adult cell proliferation and neurogenesis were assessed (Aimone et al. 2014). The left and right SVZ were isolated and combined, and RT-qPCR was utilized to determine the expression levels of the following neuronal markers: (i) brain-derived neurotrophic factor (BDNF), a crucial player in proliferation, differentiation, and function of precursor cells in adult mice (Bath et al. 2012) in which cells localized in the SVZ have been shown to be highly responsive to BDNF (Zigova et al. 1998); (ii) Bmi-1, a polycomb family transcriptional repressor that is required for the maintenance of neural stem cells in the SVZ (van der Lugt et al. 1994) and has been shown to promote neurogenesis in vivo (Mich et al. 2014); and (iii) Sox2, a transcription factor that has been found to be highly important in the regulation of self-renewal and homeostasis of neural stem cells in the SVZ (Episkopou 2005). Sox2 has also been shown to be expressed in proliferating precursor cells and critical for the survival of these cells (Feng et al. 2013). In addition to these markers, expression levels of nestin, an intermediate filament specifically expressed by newborn neural stem cells in the adult nervous system, and DCx were assessed to evaluate alignment with marked proteins observed in the fluorescent IHC. Stimulated mice showed significantly upregulated expression in BDNF, Bmi-1, and Sox2 (Fig. 6c), indicating that treatment can affect key factors in the SVZ involved in neurogenic processes. No differences were observed in DCx, and the approximate 1-fold increase in nestin expression was not significant (*P* = 0.0505).

## Discussion

In this study, we demonstrated that precisely timed, task-matched DBS of the NAc can augment spatial memory recovery after TBI in a mouse HPC contusion model. Mice that received DBS learned at a faster rate and to a greater extent than untreated injured mice and to a level comparable to uninjured control mice. These effects persisted after stimulation cessation and a 10-day rest period, demonstrating that stimulation augmented memory and resulted in retained effects. Additionally, treated mice exhibited greater cellular adaptation and had upregulation of genes associated with neural differentiation, migration, cell signaling, and neural proliferation.

Following TBI, many individuals are left with substantial deficits in cognitive domains, including learning and memory (Murray et al. 1999; Myburgh et al. 2008; Rabinowitz and Levin 2014). We have demonstrated in this work that mice receiving unilateral stimulation of the NAc exhibited greater behavioral performance improvement than untreated control mice, at a level approximating that of uninjured control mice and retained their performance gains long after stimulation was halted, suggesting that stimulation induced long-term adaption. These findings suggest that precisely timed, task-matched stimulation of the NAc may be an efficacious treatment to augment functional outcomes after TBI. Two prior studies of DBS for TBI in rodent models have used open-loop DBS but did not demonstrate sustained improvement in performance following the cessation of treatment and did not target relevant task epochs (Carballosa Gonzalez et al. 2013; Lee et al. 2013).

The NAc is believed to be an essential node in the learning and memory system with extensive connectivity (Mizumori et al. 2004). The NAc receives input from the hippocampal formation and the midbrain dopaminergic system, allowing memory and reinforcement information to converge (Groenewegen et al. 1987; Fallon 1988). Furthermore, output projections from the NAc allow direct and indirect influence on learning centers in prefrontal cortex and motor execution centers in the brainstem (Berendse et al. 1992), facilitating the integration of learning with motor action (Brown and Sharp 1995). Specific to spatial learning, the NAc plays a role in translating hippocampal spatial input into weighed sensorimotor sequences, incorporating path information from prefrontal cortex and motivational signals from the amygdala (Poucet et al. 2004). Under this framework, stimulating when the animal encountered the designated reward location could work to enhance active networks, reinforcing processes of synaptic efficacy and altering the signal to noise ratio of the spatial learning and memory circuitry.

Spatial memorization during the reinforcement period relies on early activation of synaptic receptors, which induces structural modifications in pre- and postsynaptic neurons that alter synaptic function (Rinaldi et al. 2012). In line with this, overexpression of synapsin-1 and GAP43 has been shown to enhance neurotransmitter release and terminal remodeling (De Graan et al. 1990; Holahan et al. 2007). Therefore, the promotion of these proteins with striatal stimulation could enhance signal propagation through the striatum and hippocampus, leading to enhanced spatial memory function. In this study, synapsin-1 expression was diminished in untreated injured animals when compared to treated and control mice. Previous studies have demonstrated similar results (Hardman et al. 1997), likely due to axonal diffusion caused by unilateral ablation of hippocampal connections (McGeorge and Faull 1989). GAP43 was similarly diminished in untreated injured mice aligning with previous studies that have shown increased potentiation in the HPC for naïve, healthy mice during early spatial memory learning (Wilson and Tonegawa 1997; Pascale et al. 2004). In line with these findings, experience-dependent synaptic plasticity in the NAc is believed to be responsible for long-term stabilization of spatial information (Di Chiara et al. 2004). The synapsin-1 and GAP43 analysis indicate that unilateral DBS in the NAc augmented synaptic density and promoted neural outgrowth bilaterally in injured mice in both the NAc and HPC, suggesting a potential mechanism for the enhanced learning and long-term memory benefits observed during behavioral testing. Notably, the temporal specificity of the DBS strategy was paramount, as continuous stimulation and stimulation at different time points did not elicit the same effect. Temporally specific electrical stimulation can effect rapid modulation of synaptic strength, altering both synaptic response and the synaptic memory of recent history (Dobrunz and Stevens 1999). Striatal stimulation has also been shown to modulate neurotransmitter release (Collingridge and Davies 1981; Gale et al. 2013). Our histology and genomic data also reveal that stimulation induces multifaceted effects on different timescales.

Immunohistochemistry analysis of stimulated animals revealed a considerable increase in neural progenitor cells in the SVZ, which is supported by the upregulated gene expression of key factors necessary for neurogenesis as well as by a substantial increase in growth cones and synaptic density in both the HPC and NAc. This is not surprising, as brain injury has been shown to activate neuroregenerative mechanisms (Rice et al. 2003; Kernie and Parent 2010); however, treated mice showed significantly greater BrdU incorporation compared to untreated mice. These results indicate an enhanced presence of neural progenitor cells, likely through promotion of cell proliferation or prolonged neuronal survival. Interestingly, increased labeling in injured mice was also seen bilaterally, with significantly greater BrdU incorporation for treated mice compared to untreated and control mice on the contralesional side (Fig. 6b, left), suggesting that unilateral stimulation caused bihemispheric effects. We found similar results bilaterally in the RMS, where all injured mice showed an increase in BrdU incorporation when compared to control mice; however, treated mice again demonstrated significantly more incorporation than both untreated and control mice. This finding may imply that stimulation can accelerate or preserve the migration of newly generated neurons, a potentially important mechanism for augmenting brain repair following TBI. Incorporation of BrdU was also found in the HPC of all mice (Fig. 6b, left), as would be expected after spatial memory learning (Gould et al. 1999); however, there were no significant differences between cohorts, aligning with previous findings that have demonstrated that striatal stimulation does not induce hippocampal neurogenesis (Winter et al. 2015). These observations may be explained by stimulation in the NAc having not only a local effect that can modulate brain regions through the dopaminergic system but also the ability to modulate widespread changes through antidromic activation of corticostriatal connections (McCracken and Grace 2009; Albaugh et al. 2016). This highlights the influence of the NAc as a central node in learning and memory, as stimulation of other subcortical structures, such as the anterior thalamic nucleus, has not been shown to have the same effect (Encinas et al. 2011). We also observed a strong promotional effect in the contralesional cortex, indicating that unilateral stimulation can affect interhemispheric interactions to modulate the balance of bilateral cortical recovery (Albaugh et al. 2016). A similar finding was reported in the motor cortex in a stroke-induced rodent model (Kleim et al. 2003; Plautz et al. 2003).

Gene expression analysis revealed broad upregulation of genes, including BDNF and Bmi-1, suggesting enhanced neural proliferation. Overall, gene expression analysis in the SVZ supported immunohistochemistry findings of increased neurogenic activity, demonstrating that markers needed for neural stem cell maintenance, survival, and proliferation in the SVZ are augmented in animals that received stimulation during behavioral testing. Although both nestin and DCx expression levels were not found to be significantly different between the 2 populations, these results may indicate a misalignment in timing between already synthesized proteins marked in IHC and RNA levels quantified by qPCR. These findings suggest that stimulation in the NAc can augment mechanisms at both the molecular and systems levels.

This work demonstrates that temporally precise activation of the NAc can augment intrinsic neuronal mechanisms and maximize behavioral outcomes compromised by TBI. Although the result was specific to a spatial memory task, RL can be implemented in a multitude of ways to enhance different aspects of cognition. Additionally, the widespread acceptance of DBS for other indications in humans makes NAc DBS a particularly exciting and plausible intervention. The rich connectivity of the NAc has implicated this region in a number of learning, memory, and motivational processes (Bichot et al. 2011; Clithero et al. 2011), which are key components of cognitive and motor recovery. Furthermore, this study demonstrates that stimu-lation in the NAc can enhance healing and protective mechanisms, which may benefit the neural recovery process in general. Accordingly, NAc microstimulation for the treatment of cognitive dysfunction in other brain injuries, and those with more diffuse anatomical injuries, may also prove beneficial. It is also noteworthy that our study was executed in a subacute phase of injury, not limiting the benefits of treatment to acute care. Our findings demonstrate that the postinjury period represents a major and underutilized opportunity to apply neuromodulatory interventions to optimize functional recovery. The broad control experiments performed, using multiple stimulation paradigms and stimulation targets, suggest that these results are highly specific to the studied intervention.

This study is subject to a number of limitations. While the behavioral results are highly encouraging, the stimulation paradigm would need to be adapted for human subjects. There is also a need to evaluate neuronal precursors for longer than 5 days to elucidate survival of increased progenitor cells and identify cell differentiation. In line with this, evaluating where neuronal precursors migrate to and what they become will be critical to clarifying whether DBS is a viable treatment for driving intrinsic brain self-repair mechanisms. Additionally, while neural proliferation contributes to TBI recovery and may be augmented by DBS, the behavioral improvements observed in this study take place too early to suggest this as the underlying mechanism. The stimulation paradigm used in this study was unilateral (ipsilateral to the side of injury); future work investigating bilateral stimulation for the treatment of cognitive dysfunction, particularly in diseases with bilateral brain damage, may allow for further optimization.

While preliminary, our study provides a comprehensive reference for the effects of NAc DBS in the context of TBI. Our findings demonstrate that DBS can influence cellular and molecular processes, providing a tool that can manipulate adaptation in the brain to reveal key mechanisms underlying cognitive recovery. Identification of such factors can then inform research focused on targeting interventions, such as gene therapy, that hinge upon identified biomarkers pivotal for neuroregeneration and repair.

## Conclusion

This study demonstrates a novel application of task-matched stimulation of the NAc to enhance behavioral rehabilitation and augment neural recovery in TBI. The observed behavioral effects persisted after stimulation was ceased, suggesting that stimulation-induced long-term benefits. Additionally, there was greater cellular adaptation and increased expression of genes associated with neural proliferation. These results provide encouraging early evidence for the potential of NAc DBS to improve functional outcomes following TBI.

## Supplementary Material

Final_NAcc_Stim_Supplement_tgac016

## Data Availability

The data supporting the findings of this study are available from the corresponding author upon reasonable request.
