# Supplementary material for: Phasic stimulation in the nucleus accumbens enhances learning after traumatic brain injury
Source: Cereb Cortex Commun. 2022 Apr 9;3(2):tgac016. doi: 10.1093/texcom/tgac016 (PMC9070350; doi:10.1093/texcom/tgac016)
Supplement: Final_NAcc_Stim_Supplement_tgac016 [file final_nacc_stim_supplement_tgac016.docx]

**Supplemental Material**

**Supplementary Methods**

***Wire-grip test:*** The wire-grip test was conducted to establish baseline motor function. Animals were placed at the center of an 18-gauge wire suspended taut between two poles 20cm above the ground. The degree of attachment and movement of the mouse were scored. A score of 0 was given if the mouse fell within 30 seconds, 1 point for grasp with a single extremity, 2 points for grasping with multiple extremities, 3 points for grasp with multiple extremities and the tail, 4 points for moving along the wire to the pole, and 5 points for climbing down the pole within 60 seconds. Animals were tested on post-operative days 3, 5, and 7 after CCI, as well as post-operative day 4 after electrode placement, and after the first 5 days of MWM testing. Injured animals were divided into treated (receive stimulation during testing) and untreated (did not receive stimulation during testing) based on their average wire grip scores. The average test score for each animal was calculated, and each group was determined such that the total average across all animals in each group was equivalent. Two-tailed Student’s t-tests were used to compare performance between groups on the wire grip tests. This ensured that treated and untreated groups were comprised of animals with equally assessed motor impairment.

***Real-time polymerase chain reaction primers:*** Primer sequences used are as follows: Nestin F: 5’-CTCAACCCTCACCACTCTATTT-3’; Nestin R: 5’-CTGTGTCCAGACCACTTTCTT-3’; Sox-2 F: 5’-CGAGATAAACATGGCAATCAAATG-3’; Sox-2 R: 5’-AACGTTTGCCTTAAACAAGACCAC-3’; Dcx F: 5’- CCTACACCTCTTGGCTCTTATTC-3’; Dcx R: 5’-GTGGAGTTCAGCGAGCTATTT -3’; BDNF F: 5’- GGTCACAGCGGCAGATAAAAAG-3’; BDNF R: 5’- TGAATCGCCAGCCAATTCTC-3’; Bmi-1 F: 5’-CCAATGGCTCCAATGAAGACC-3’; Bmi-1 R: 5’- TTGCTGCTGGGCATCGTAAG-3’; GAPDH F: 5′-ATGACATCAAGAAGGTGGTG-3′; and GAPDH R: 5′-CATACCAGGAAATGAGCTTG-3′.

**Supplementary Figures**

**
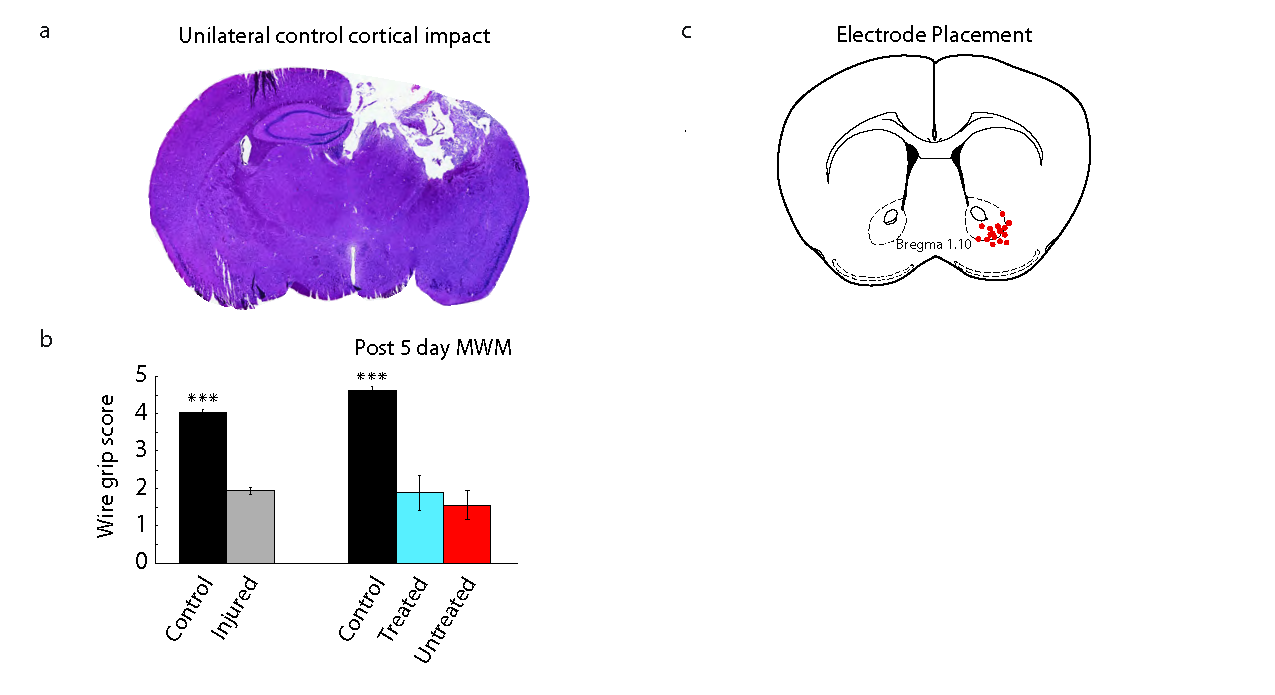
**

**Supplementary Figure 1. Traumatic brain injury assessment. a**, Representative H&E coronal section demonstrating damage from cortical impact. The section shows the complete unilateral destruction of the anterior hippocampus. **b**, *(left)* Wire grip testing scores post-injury to assess basic anxiety and motor damage caused by impact. Uninjured control animals (n= 22) had significantly higher testing scores demonstrating motor skills to securely grip the wire and cognitive awareness to traverse the wire to safety. Injured animals (n= 43) received approximately half the score of control animals indicating a slight impairment in motor skills to grip the wire and impairment in cognitive skills and anxiety demonstrated as a result of not traversing the wire. *(right)* Comparison of wire grip testing scores after 5 days of MWM testing demonstrated no major change in scores for uninjured (n= 6), treated (n= 6), and untreated (n= 6) animals. A significant difference in performance was found between the control and both treated and untreated animals respectively ^*^*p*<0.001. *P*-values calculated using a two-tailed Student’s t-test.

**
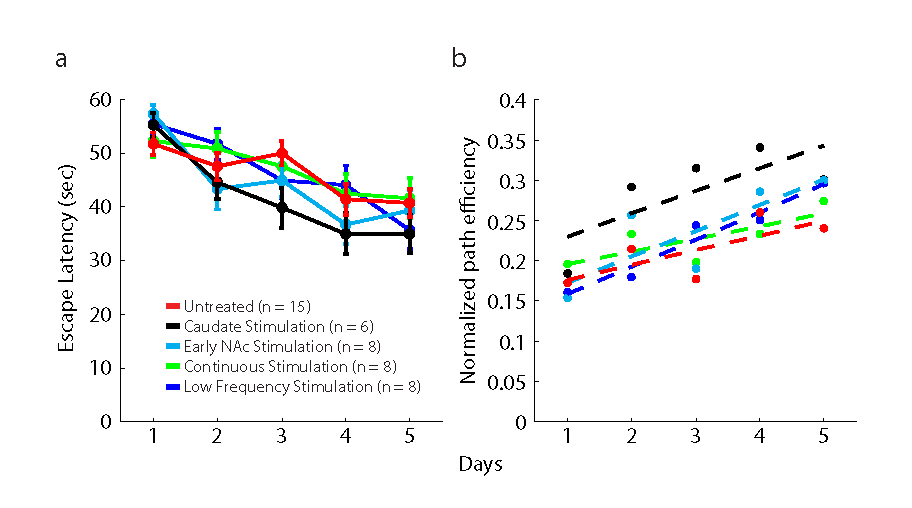
**

**Supplementary Figure 2. Stimulation control testing. a**, Escape latency decreased across all tested groups, but there were no statistically significant differences between groups (repeated measure ANOVA). Phasic stimulation was applied in the caudate nucleus upon arrival on the platform (n= 6 animals), testing the importance of targeting the NAc role in the spatial memory task. Low-frequency phasic stimulation was applied in NAc upon arrival on the platform (n= 8 animals), testing the importance of inducing activation with appropriate stimulation parameters. Stimulation was applied continuously in the NAc during each trial of the task (n= 8 animals), testing the importance of applying brief, precisely timed stimulation that attempts to induce activation during relevant time points. Phasic stimulation was applied in the NAc at the beginning of the trial (n= 8 animals), testing the importance of temporal specificity. **b**, Path efficiency increased in all groups across five days of testing with no significant difference in the rate of rise between groups when comparing distributions, or the distribution of slope coefficients for each group output from the linear regression of path efficiency across days for each animal (Student’s two-tailed t-test). Values are mean ± s.e.m. or mean alone.

**
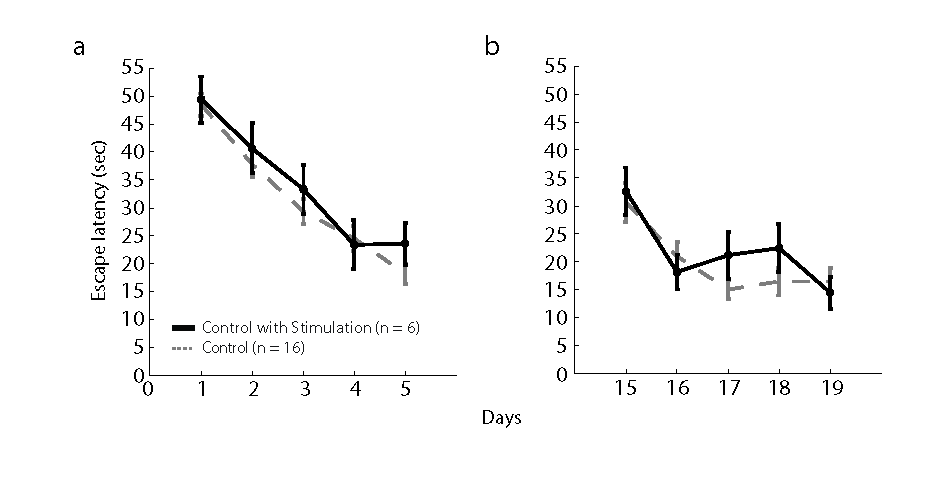
**

**Supplementary Figure 3. Uninjured animal performance with stimulation. a**, Escape latency decreased in both uninjured control animals that received stimulation and uninjured animals that did not receive stimulation during the first five days of testing. Stimulation was applied to the NAc upon arrival on the platform. Stimulated control animals (black, n= 6) showed no difference in performance compared to untreated control animals (dashed gray, n= 16; repeated measure ANOVA). **b**, After the first five days of testing, animals in each group were given ten days’ rest before being re-tested. Again, a repeated measure ANOVA did not identify any differences between stimulated animals (black, n= 6) and unstimulated controls (dashed grey, n= 11). Values are mean ± s.e.m.


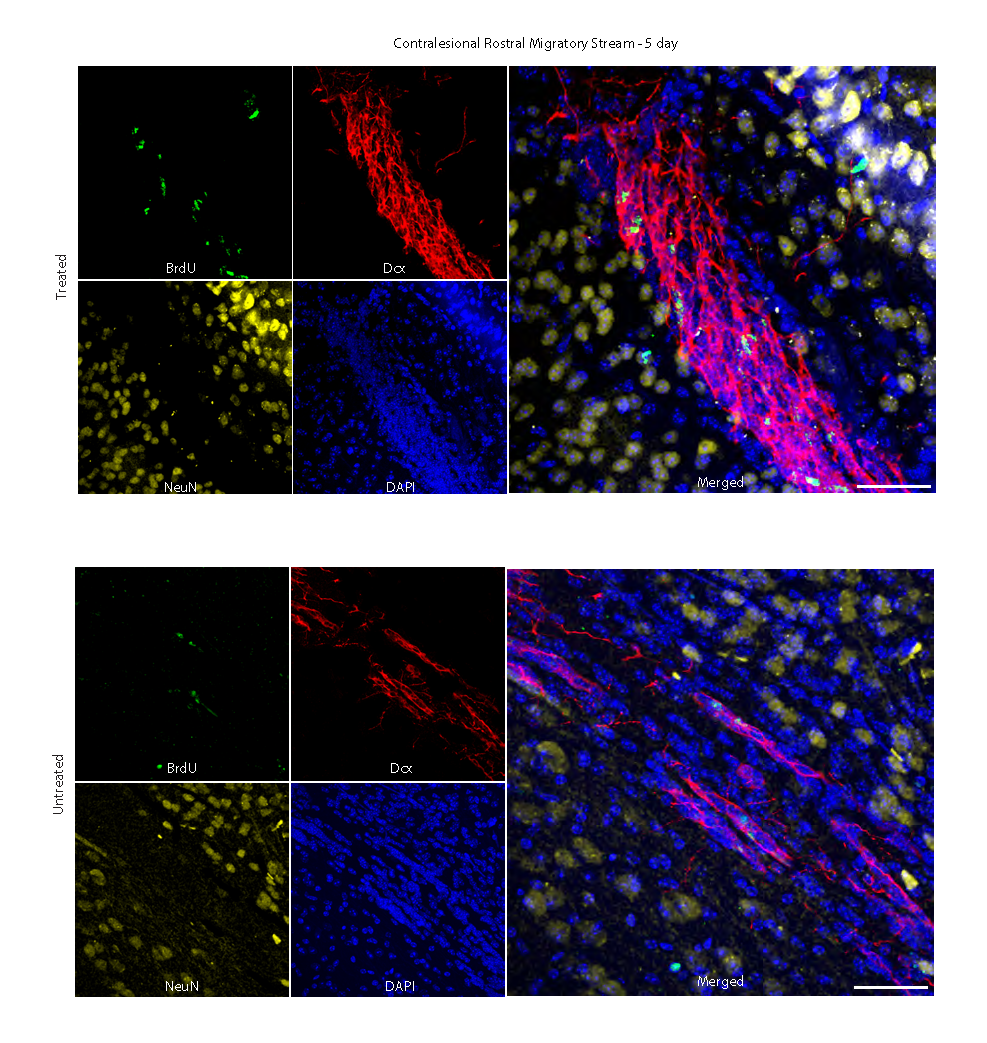


**Supplementary Figure 4. Stimulation-induced increase of neural progenitor migration.** Representative images, taken by a confocal 40x oil immersed objective, showing contralesional RMS in treated (top) and untreated (bottom) animals, respectively, after 5 days of MWM testing. Expression of co-labeled Dcx (red) and BrdU (green) qualitatively demonstrate the increase of neural progenitor cells along the RMS of treated animals. Scale bars = 50µm.
